# A neutrophil extracellular traps-associated lncRNA signature predicts the clinical outcomes in patients with lung adenocarcinoma

**DOI:** 10.3389/fgene.2022.1047231

**Published:** 2022-11-07

**Authors:** Wencong Ding, Biyi Li, Yuan Zhang, Liu He, Junwei Su

**Affiliations:** ^1^ Department of Rheumatology, Affiliated Guangdong Hospital of Integrated Traditional Chinese and Western Medicine of Guangzhou University of Chinese Medicine, Foshan, Guangdong, China; ^2^ Department of Emergency Foshan Hospital of Traditional Chinese Medicine, Foshan, Guangdong, China; ^3^ Department of Emergency, Affiliated Guangdong Hospital of Integrated Traditional Chinese and Western Medicine of Guangzhou University of Chinese Medicine, Foshan, Guangdong, China; ^4^ Guangdong Hospital of Traditional Chinese Medicine, Guangzhou, Guangdong, China

**Keywords:** neutrophil extracellular traps (NETs), long non-coding RNAs (lncRNAs), risk score, lung adenocarcinoma (LUAD), tumor mutation burden

## Abstract

**Backgrounds:** Neutrophil extracellular traps (NETs) play an important role in the occurrence, metastasis, and immune escape of cancers. We aim to investigate Long non-coding RNAs (lncRNAs) that are correlated to NETs to find some potentially useful biomarkers for lung adenocarcinoma (LUAD), and to explore their correlations with immunotherapy and chemotherapy, as well as the tumor microenvironment.

**Methods:** Based on the The Cancer Genome Atlas (TCGA) database, we identified the prognosis-related lncRNAs which are associated with NETs using cox regression. The patients were then separated into two clusters based on the expression of NETs-associated lncRNAs to perform tumor microenvironment analysis and immune-checkpoint analysis. Least absolute shrinkage and selection operator (LASSO) regression was then performed to establish a prognostic signature. Furthermore, nomogram analysis, tumor mutation burden analysis, immune infiltration analysis, as well as drug sensitivity analysis were performed to test the signature.

**Results:** Using univariate cox regression, we found 10 NETs-associated lncRNAs that are associated with the outcomes of LUAD patients. Also, further analysis which separated the patients into 2 clusters showed that the 10 lncRNAs had significant correlations with the tumor microenvironment. Using LASSO regression, we finally constructed a signature to predict the outcomes of the patients based on 4 NETs-associated lncRNAs. The 4 NETs-associated lncRNAs were namely SIRLNT, AL365181.3, FAM83A-AS1, and AJ003147.2. Using Kaplan-Meier (K-M) analysis, we found that the risk model was strongly associated with the survival outcomes of the patients both in the training group and in the validation group 1 and 2 (*p* < 0.001, *p* = 0.026, and *p* < 0.01). Using receiver operating characteristic (ROC) curve, we tested the sensitivity combined with the specificity of the model and found that the risk model had a satisfactory level of 1-year, 3-year, and 5-year concordance index (C-index) (C = 0.661 in the training group, C = 0.679 in validation group 1, C = 0.692 in validation group 2). We also explored the immune microenvironment and immune checkpoint correlation of the risk model and found some significant results.

**Conclusion:** We constructed a NETs-associated lncRNA signature to predict the outcome of patients with LUAD, which is associated with immunephenoscores and immune checkpoint-gene expression.

## Introduction

Lung Adenocarcinoma (LUAD) is a particular pathologic type of non-small cell lung cancer (NSCLC), which accounted for nearly 90% of deaths of lung cancer worldwide (Relli et al., 2019). As the most common primary lung cancer type, LUAD is mainly caused by tobacco smoking—whether primary or secondary exposure, indoor/outdoor air pollution, and occupational exposure to other harmful agents such as silica, asbestos, radon, heavy metals, and so on. Despite all those reasons, the reason ranked first in etiology in LUAD is tobacco smoking (Hutchinson et al., 2019). Traditional treatments of LUAD include surgical excision, chemotherapy, and radiotherapy. Newly discovered treatment therapies, for example, immunotherapy is also making progress in the treatment of LUAD (Succony et al., 2021). Presently, evidences have shown that the discovery and application of new molecular biomarkers is quite promising in improving the outcomes of patients with LUAD (Xu et al., 2020).

Neutrophils play an indispensable role in the immune response. Neutrophil extracellular traps (NETs) are structures released by immune cells under various stimulations or pathological conditions (Kolaczkowska and Kubes, 2013; Németh et al., 2016). NETs are extracellular structures made up of mitochondrial and nuclear DNA as well as histones, which have been recently considered an innate defense mechanism to constrain and eliminate invading pathogens (Pruchniak and Demkow, 2019). The process of classical NETs formation is defined as “neutrophil extracellular traposis (NETosis)”, which has been identified as a unique form of regulated cell death, which is different from programmed-cell deaths, such as apoptosis, ferroptosis, and pyroptosis (Manda-Handzlik et al., 2020). NETs play a vital role in the development and progression of tumors. Although the NET is considered an immune response against pathological conditions, there are still a lot of researchers who claim otherwise. In hepatocellular carcinoma (HCC), NETs were proved to induce the metastasis of primary HCC (Dickson, 2020; Yang et al., 2020). Also, NETs could render the metastasis of breast cancer, which was induced by cancer cells (Park et al., 2016). Therefore, it is essential to find out the important biomarkers related to NETs to predict the prognosis of LUAD, and to provide possible therapeutic targets for this disease (Mutua and Gershwin, 2021).

Long non-coding RNAs (lncRNAs) are RNAs with a length of more than 200 nucleotides that do not have the function of encoding proteins and play important roles in a wide range of cellular processes (Eptaminitaki et al., 2022). By participating in pathophysiological activities such as cell growth, apoptosis, invasion and metastasis, lncRNAs play a key regulatory role in the development and evolution of cancers, so it can be used as a tumor marker for a variety of malignant tumors, including LUAD (Vallone et al., 2018; Tian et al., 2020; Tian et al., 2021). In addition, multiple lncRNAs have been identified as promising biological therapeutic targets and closely related to drug resistance of lung cancer (Chen et al., 2020; Yu et al., 2020; Zhang et al., 2021). Also, studies related to the mechanisms regarding the synergetic interactions between those NETs associated lncRNAs are becoming more and more important (Fang et al., 2021).

In this article, we constructed a NETs-associated lncRNA risk model for the prediction of prognosis based on public databases and repositories. Kaplan-Meier survival analysis and ROC analysis were used to assess the validity of the model. Also, based on the risk model we acquired, analyses were employed to investigate the relationship between the model and tumor immunity, immune checkpoint, and chemotherapeutic sensitivity.

## Materials and methods

### Data acquisition and processing

LUAD patients’ transcriptomic data and clinical information were downloaded from TCGA database (LUAD samples: 539, normal samples: 59) (Tomczak et al., 2015). Samples with no follow-up information and incomplete clinical information were also deleted, 478 tumors samples were retained for this study. Perl software was used to integrate the raw data into an expression matrix.

### NETs associated-lncRNA downloading and acquisition

We identified 469 lncRNAs that had close correlation with NET-related genes from the TCGA database based on Pearson analysis and he standard used in this part was Pearson R > 0.5 and *p* < 0.001 (Liu et al., 2022).

### Survival analysis using univariate cox regression

Univariate cox regression was conducted for lncRNAs that are identified as NETs-related lncRNA associated with prognosis using the R software package *survival* (version 3.2)*.* The lncRNAs with survival significance (*p* < 0.01) were filtered to conduct further analysis.

### Consensus clustering analysis

To evaluate the characteristics of classifying patterns of NETs in prognosis and immune feature, all LUAD patients were divided into two subgroups by performing consensus clustering analysis. This method identified distinct NETs modification patterns based on the expression level of NETs-related genes by “ConsensusClusterPlus” package. For the major parameters in the “ConsensusClusterPlus” function, the following was set: the max cluster number (maxK) = 9, proportion of items to sample (pItem) = 0.8, proportion of features to sample (pFeature) = 1, cluster algorithm (clusterAlg) = hc/hierarchical, and distance = spearman. The above process is repeated 1,000 times to ensure the consistency of the classification (Wilkerson and Hayes, 2010).

### Establishing a prognostic signature using LASSO regression

We randomly divided the entire set (478 samples) into two sets using the R package “caret.” The least absolute shrinkage and selection operator (LASSO) regression was performed to construct a prognostic signature to reduce the number of variables and to reduce Multicollinearity in our model. The risk score can be illustrated as follows: Risk score = $\overset{n}{\underset{i=1}{\sum }}\beta_{i}*{gene\_expression}_{i}$ *(β*_*i*_ *: coefficient of gene i; gene_expression*_*i*_ *: expression of gene i).* The patients were grouped by risk scores, which divided them into high-risk and low-risk groups (Ni et al., 2022). Survival analysis was performed accordingly using package *Survival* (version 3.2).

### Time ROC curve analysis for assessing the prognostic ability of the model

The receiver operating characteristic (ROC) curve is a graphical plot that illustrates the diagnostic ability of a binary classifier system as its discrimination threshold is varied (Hoo et al., 2017).

### Tumor immune infiltration analysis

In this study, we employed multiple methods for tumor immune infiltration, including TIMER, QUANTISEQ, ESTIMATE, and so on (Li et al., 2017; Chakraborty and Hossain, 2018). Immune checkpoint analysis was also performed to examine the immunological differences between the high-risk and the low-risk groups. The details of the immune-infiltration analysis have been intensively described in our previous works (Liu et al., 2021a).

### Tumor mutation burden analysis

Tumor mutation burden (TMB) analysis, which refers to the density of non-synonymous mutation in the protein-coding area of the tumor cell genomes (Cui et al., 2021).

### Nomogram construction

The nomogram of the risk score and relevant clinical information was depicted using package *survival* (version 3.2) and package *RMS* (version 6.3) (Wu et al., 2020).

### Immunophenoscore analysis

Immunophenoscore (IPS) consists of MHC molecular (MHC), effector cells (ECs), immune checkpoints (CPs), and immunosuppressive cells (SCs). Immunophenotype scores with a scale ranging from 0 to 10 was calculated using the expression of representative genes or immune manifestation of gene sets (Xu et al., 2021). The IPS of LUAD patients were obtained from the Cancer Immunome Atlas (TCIA) framework (Kirby et al., 2020). Furthermore, IPS z-score is regarded as an integration of the four phenotypes: MHC, CPs, SCs and ECs.

### Tumor stemness analysis using stemness scores

To analyze the features of tumor stem cells in LUAD patients, we downloaded RNA expression data and DNA methylation data for LUAD from TCGA. RNA stemness score (RNAss) and DNA stemness score (DNAss) of the patients were presented using the R packages “limma” and “corrplot” correspondingly (Zhang et al., 2020). The algorithms for calculation of tumor stemness have been introduced and described by scientists previously (Malta et al., 2018).

### Drug sensitivity analysis

The “*pRRophetic”* package and the expression matrix of LUAD patients was used for predicting the minimum drug inhibition concentration (IC_50_) of drugs in uveal melanoma patients of high-risk and low-risk groups (Geeleher et al., 2014). Drugs that have statistically different IC_50_ values and may become candidates for the treatment of LUAD were obtained as potential therapeutic drugs.

### Statistical analysis

R v.4.1.0 was used to do statistical tests. The differences of the two subgroups were calculated by Student’s t test and ANOVA. Kaplan-Meier analysis and log rank test were employed to calculate the discrepancy of OS between the two risk groups. The relationships between risk score and immune infiltration level were calculated by Pearson correlation test. *p* < 0.05 was defined to have statistical difference.

## Results

### 10 NETs-related lncRNAs were filtered as the potentially prognosis-related lncRNAs

**FIGURE 1:**
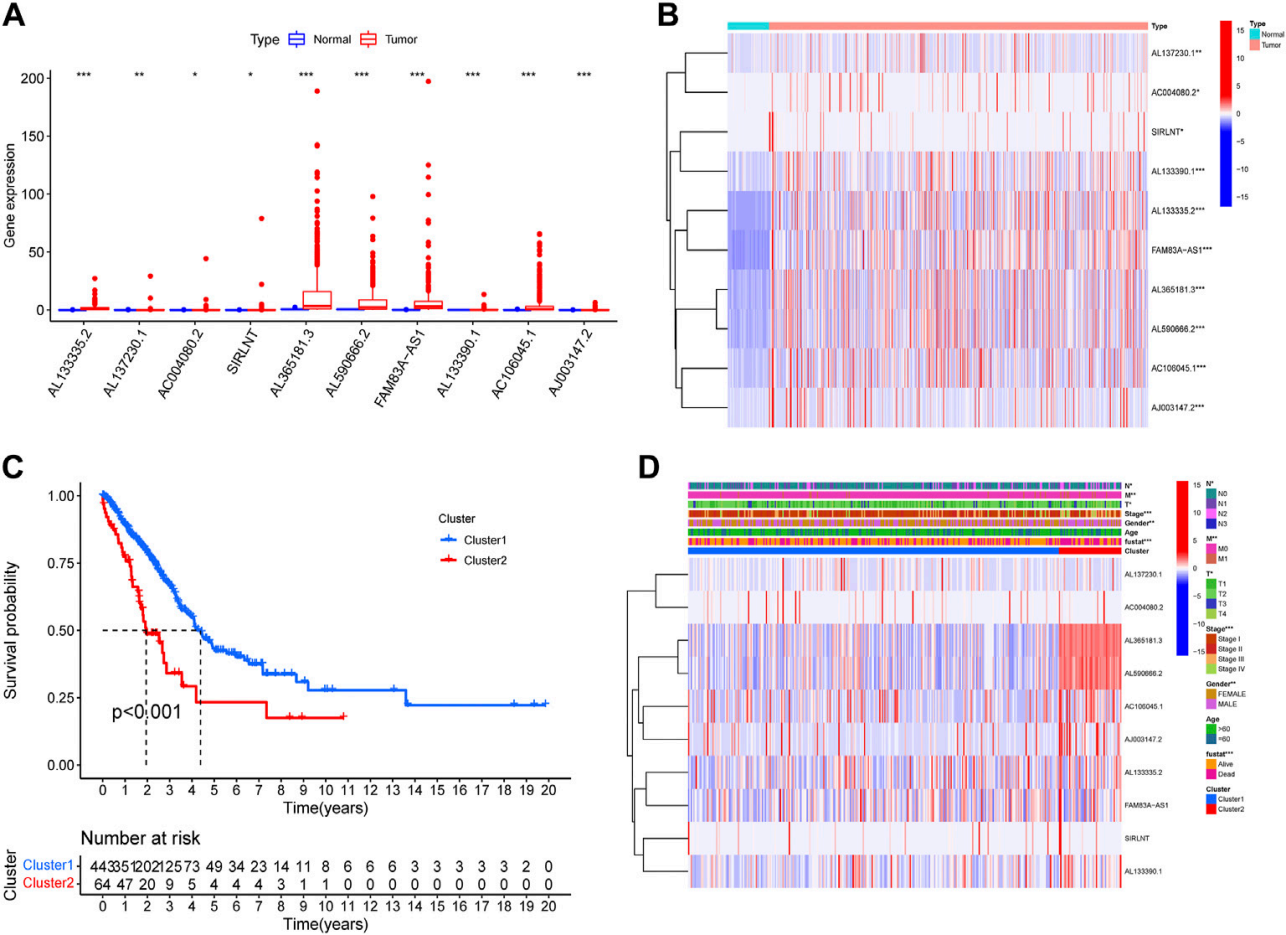
Different prognosis and clinicopathological features of two LUAD clusters. Boxplot **(A)** and heatmap **(B)** revealed the difference in expression of 10 NETs-associated lncRNA in normal and tumor tissues **(C)** The overall survival rate of LUAD patients in the two clusters was calculated by Kaplan-Meier curve **(D)** Heatmap exhibited the differences in expression of 10 NETs-associated lncRNA and clinicopathological features of two LUAD clusters (*p < 0.05, ** *p* < 0.01, *** *p* < 0.001).

We identified 469 lncRNAs that was correlated with Nets from TCGA database by Pearson correlation analysis with the correlation coefficient >0.5 and *p* < 0.001. Then, the univariate cox regression was performed for the NETs-associated lncRNAs that have potential prognostic values (*p* < 0.05). 10 NETs-associated lncRNAs in total were obtained, namely AL133335.2, AL137230.1, AC004080.2, SIRLNT, AL365181.3, AL590666.2, FAM83A-AS1, AL133390.1, AC106045.1, and AJ003147.2. All lncRNAs had HR > 1, meaning that the lncRNAs were related to a poor prognosis of LUAD (Table 1). Based on the expression profiles in TCGA database, these 10 NETs-associated lncRNAs expression were different between the LUAD and normal tissues (Figures 1A,B). According to the similarity of NETs-related genes expression level and the proportion of fuzzy clustering measurement, it was found that, when k = 2, the cluster had the best stability. Therefore, the LUAD patients were separated into 2 clusters: cluster1 and cluster 2 according to the expression of the 10 NETs-associated lncRNAs. Survival analysis using Kaplan-Meier plot showed that cluster 2 had a significantly poorer prognosis than cluster1, illustrating the possible relation of the NETs-associated lncRNAs with the clinical outcomes of LUAD patients (Figures 1C,D).

**TABLE 1:** Univariate Cox regression analysis of Nets-related lncRNAs.

ID	HR	HR.95L	HR.95H	pvalue
AL133335.2	1.09015	1.035637	1.147533	0.000974
AL137230.1	1.1239	1.051428	1.201368	0.000594
AC004080.2	1.080487	1.032997	1.13016	0.000737
SIRLNT	1.058747	1.0335	1.08461	3.55E-06
AL365181.3	1.011392	1.00652	1.016287	4.27E-06
AL590666.2	1.021009	1.010469	1.031659	8.60E-05
FAM83A-AS1	1.023362	1.014632	1.032167	1.27E-07
AL133390.1	1.327722	1.132255	1.556934	0.000485
AC106045.1	1.022584	1.011173	1.034123	9.59E-05
AJ003147.2	1.348558	1.129026	1.610778	0.000972

### The expression of 10 candidate-NETs associated lncRNAs is associated with PD-L1 expression and immune infiltration scores

**FIGURE 2:**
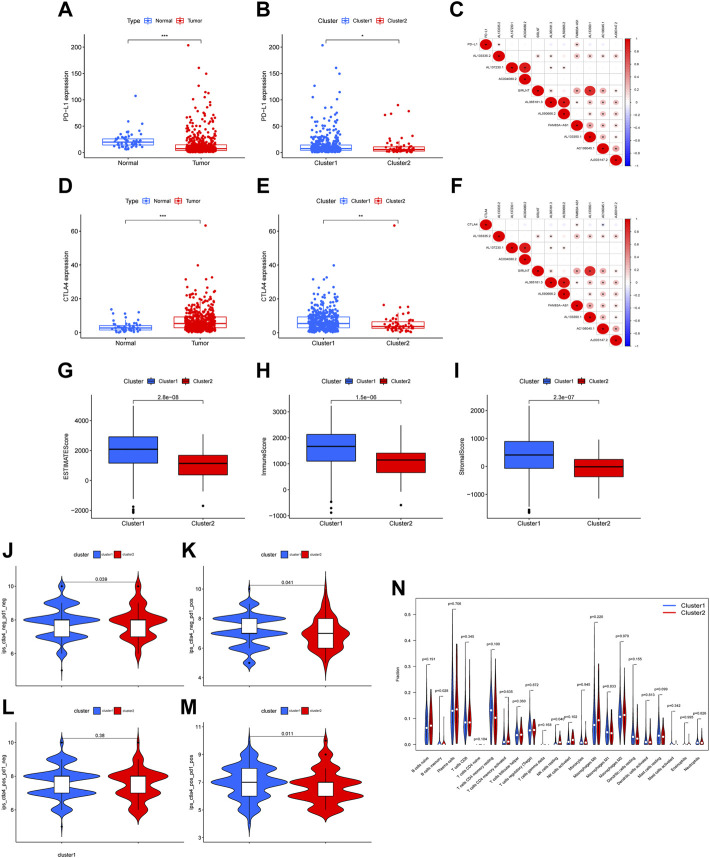
Immunoassay of two clusters. The expression level of PD-L1 between normal and tumor tissues **(A)**, between cluster 1 and cluster 2 **(B)**. The correlation between PD-L1 expression and expression of 10 NETs-associated lncRNA **(C)**. The expression level of CTLA4 between normal and tumor tissues **(D)**, between cluster 1 and cluster 2 **(E)**. The correlation between CTLA4 expression and expression of 10 NETs-associated lncRNAs **(F)**. The difference in ESTIMATE score, immune score and stromal score between cluster 1 and cluster 2 **(G–I)**. Immunephenoscores (IPS) analysis **(J–M)**. The abundance of immune infiltration cells in cluster 1 and cluster 2 **(N)** (* *p* < 0.05, ** *p* < 0.01, *** *p* < 0.001).

To further explore the immune properties of the 10 candidate-NETs associated lncRNAs, we started an analysis on tumor immune infiltration and immune checkpoint analysis. Firstly, we compared the PD-L1 and CTLA4 expression levels between the normal group and the tumor group. We eventually found that the tumor group had a lower level of PD-L1 expression compared with the normal group (Figure 2A). Similarly, the expression of PD-L1 in cluster 2 is also lower than that in cluster1 (Figure 2B). However, the expression cytotoxic T-lymphocyte-associated protein 4 (CTLA4), which is also an important immune checkpoint that could be a potential therapeutic target, is downregulated in the tumor group and upregulated in cluster 2 (Figures 2D,E). Furthermore, the ESTIMATE score, immune score, and stromal score are both lower in cluster 2 than in cluster 1, indicating the immunological differences between the two clusters (Figures 2G–I). We also performed correlation plots of the 10 candidate lncRNAs, and found a significant correlation between the expression of gene AL137230.1 and AC004080.2. Also present was a strong correlation between gene AL133390.1. Such correlation implied a potential link and interaction between those NETs-associated lncRNAs, which are worthy of our further investigations (Figures 2C,F). Furthermore, based on the expression of the PD1 and CTLA1 we categorized the patients into 4 different categories, namely CTLA4^−^PD1^-^, CTLA4^−^PD1^+^, CTLA4^+^PD1^-^, and CTLA4^+^PD1^+^. The IPS z-scores were analyzed between the 4 groups according to 4 categories. It could be noted that the distribution of the scores in cluster 1 was significantly higher than that of cluster 2 in CTLA4^−^PD1^-^, CTLA4^+^PD1^-^, and CTLA4^+^PD1^+^ patients (Figures 2J–M). The abundance of B cells memory and NK cells resting was significantly higher in cluster2 (Figure 2N).

### Construction of a prognosis-related signature of LUAD related to NETs

**FIGURE 3:**
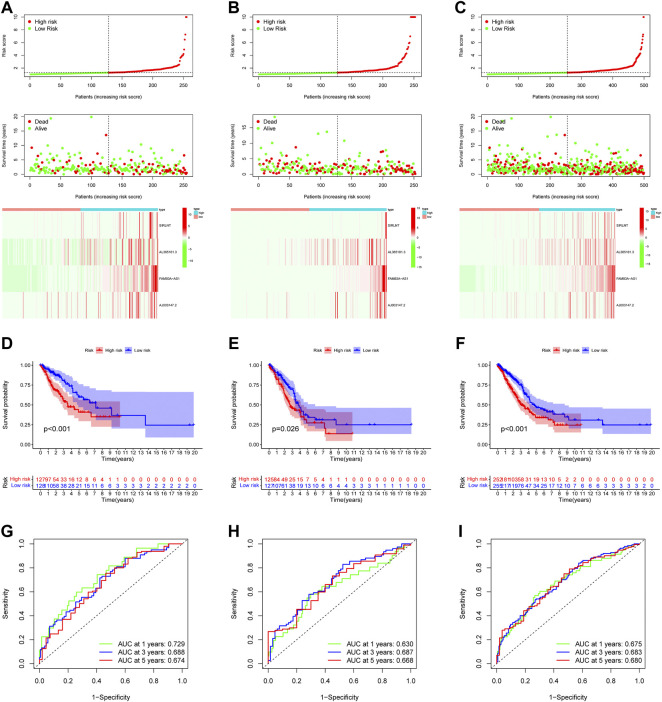
Prognostic model construction and evaluation. In the training cohort **(A)**, testing cohort **(B)** and entire cohort **(C)** the patient with different risk score, survival status, and NETs-associated lncRNAs expression were shown. Survival analysis of training cohort **(D)**, testing cohort **(E)** and entire cohort **(F)** and the prognosis of high-risk group was significantly worse. ROC curve revealed that in the training cohort **(G)**, testing cohort **(H)** and entire cohort **(I)**, the AUC values for 1, 3, and 5-year OS were over 0.6.

The strong correlation between the 10 NETs-associated lncRNAs showed significant collinearity, which meant that reducing the number of variables using methods like LASSO regression is necessary. All LUAD patients were randomly divided into training cohort, testing cohort and entire cohort, and there was no significant difference in clinical information among the three groups (Supplementary Table S1). Using LASSO regression, we found that the prognostic signature eventually contained 4 NETs-associated lncRNAs: SIRLNT, AL365181.3, FAM83A-AS1, and AJ003147.2. By setting a median risk score as the cutoff value, patients in the training group and validation group were all separated into 2 groups: the high-risk group and the low-risk group in training cohort, testing cohort and entire cohort respectively (Figures 3A–C). Survival analysis showed that the patients all showed better clinical outcomes in the low-risk group, regardless of which group they were in (Figures 3D–F). Furthermore, we constructed a time-ROC curve to evaluate the precision of the model. The 1-year, 3-year and 5-year C-index of the model in the training group were separately 0.729, 0.688, and 0.674; and the C indexes in the 2 validation groups all fell between 0.6 and 0.7, regardless of the year of the cutoff (Figures 3G–I).

### Multivariate regression validated that the risk score is independent prognostic factor

**FIGURE 4:**
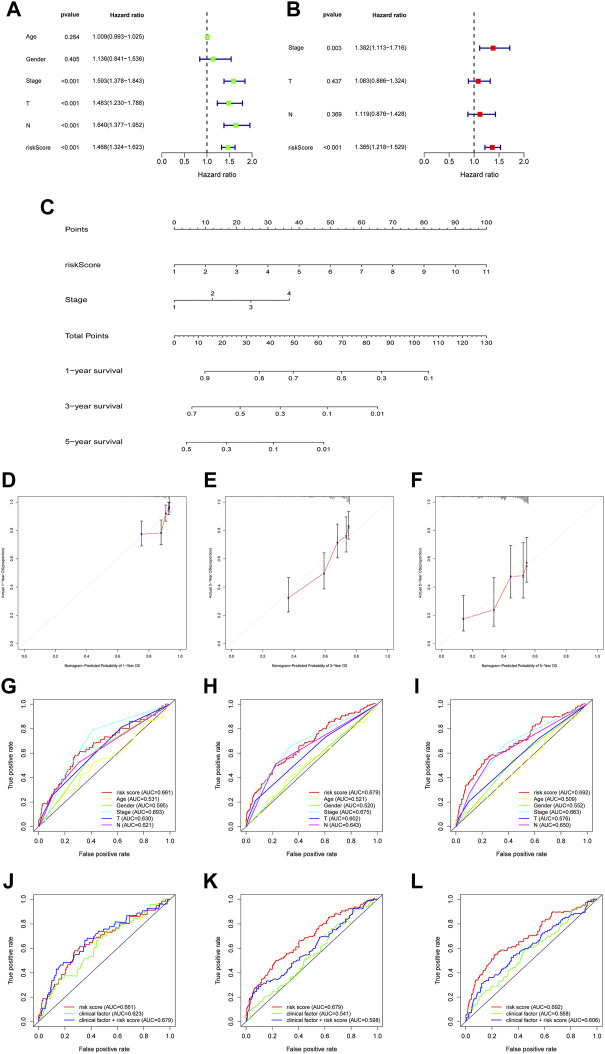
The construction and assessment of prognostic nomogram. **(A,B)** Univariate and multivariate Cox regression analyses were used to evaluate whether risk score and clinical characteristics were independent predictors. **(C)** A nomogram was constructed to predict OS **(D–F)** The calibration curves of the nomogram based on 1-, 3-, and 5-year OS **(G–I)** The ROC curve of risk score and clinical characteristics was performed based on 1-, 3-, and 5-year OS **(J–L)** When combined risk score with clinical factors for analysis, the AUC values of 1-, 3-, and 5-year OS was detected.

We next used multivariate cox regression to combine the risk score with the phenotype data to evaluate the risk score and its relevance to the patients’ clinical prognosis. Multivariate cox regression showed that the risk score had a hazard ratio of 1.466 (95%CI: 1.324–1.623) in the training group. Similarly, the hazard ratio of risk score in the validation group was 1.365 (95%CI: 1.218–1.529). The risk score in both groups had significant relevance to the clinical outcomes of the patients, which was independent of the influences of the stages, gender, age, and so on (Figures 4A,B).

### Assessment of the signature using ROC curve and nomogram

To further assess the value of the model, we used a nomogram to combine the clinical phenotypes with the risk score (Figure 4C). After combining the phenotype data with the risk score, we found that the predicted survival rate was adjacent to the actual survival rate in the comparison plot, in the entire group at 1-year, 3-year and 5-year (Figures 4D–F). Also, we compared the predicting value of the risk score with different types of clinical data, including age, gender and clinical stages, and found that the risk score demonstrated a stronger capability to predict than those clinical data (Figures 4G–I). Previous study demonstrated that as AUC >0.6, predictive signature could effectively predict the survival rate of tumor patients (Liu et al., 2022). The muti-ROC curves proved that AUC of NETs-related signature was greater than 0.6, synthesizing clinical factors and risk scores would be better than clinical factor (Figures 4J–L). Based on above results, we inferred that risk score evaluated by NETs-related genes can accurately forecast the prognosis of LUAD patients.

### The gene signature is correlated to cell-cycle and once-immunological properties

**FIGURE 5:**
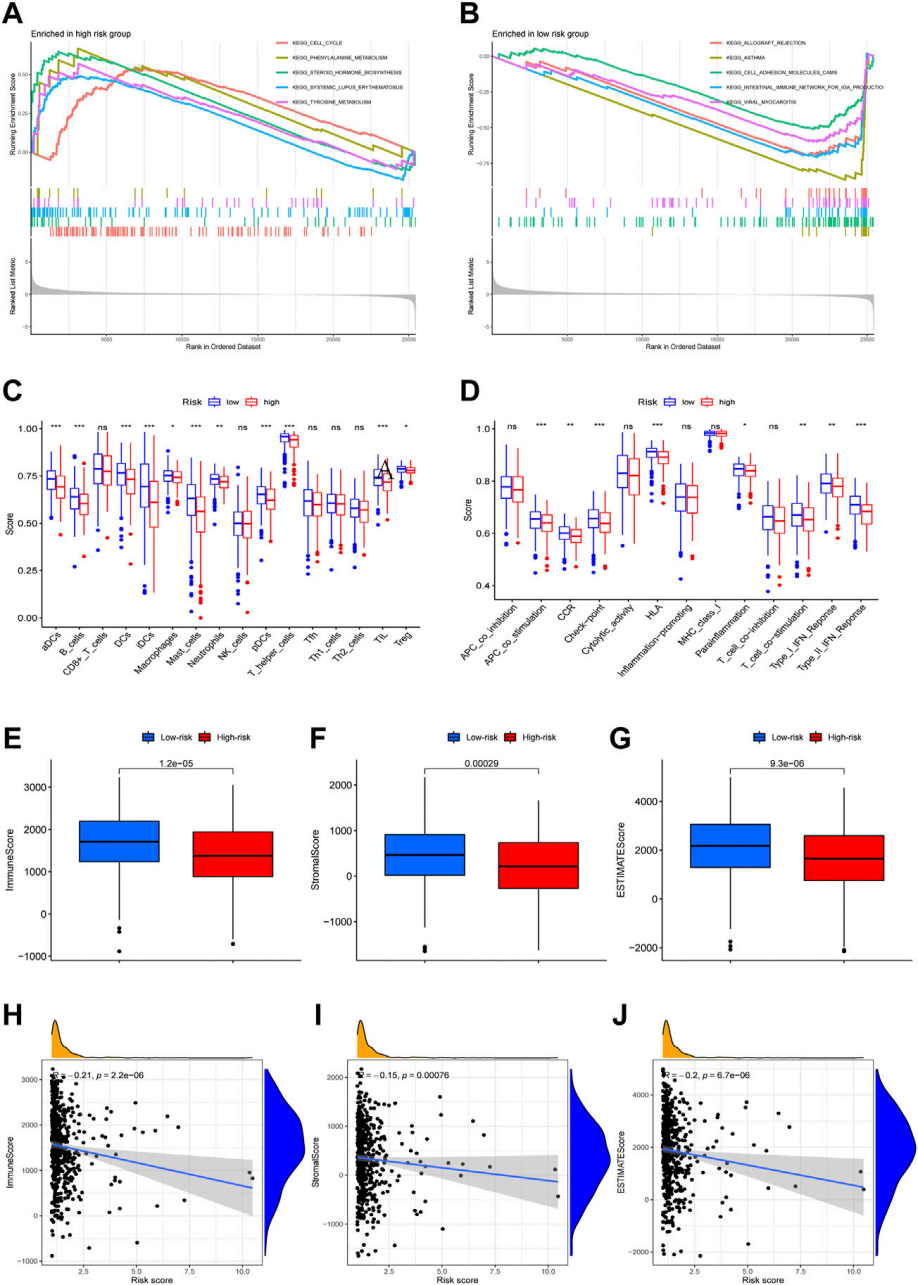
GSEA, ssGSEA and ESTIMATE analysis. Gene set enrichment analysis of Kyoto Encyclopedia of Genes and Genomes (KEGG) in high-risk and low-risk groups **(A,B)**. ssGSEA analysis showed the difference of immune cells and immune function between high-risk and low-risk groups **(C,D)**. The difference of immune score, stromal score, ESTIMATE score between high-risk and low-risk groups **(E–G)**. The correlation of immune score, stromal score, ESTIMATE score and risk score **(H–J)**. (ns, not significant, * *p* < 0.05, ** *p* < 0.01, *** *p* < 0.001).

GSEA enrichment analysis related to the gene signature showed that the pathways enriched in the high-risk group were cell cycle, phenylalanine metabolism, steroid hormone biosynthesis, and systemic lupus erythematosus (SLE). Conversely, the pathways enriched in the low-risk group were allograft rejection, asthma, cell adhesion molecules (CAMs), and intestinal immune network (Figures 5A,B). The results showed that immunological differences may be a major protection factor in the low-risk group. Immune infiltration scoring and cell components showed that the infiltration of immune cells in the high-risk group was generally lower than the low-risk group. Scorings of immunological processes also revealed that the high-risk group was lower in multiple immunological signs of progress, such as APC and T cell co-stimulation, HLA activity, checkpoint, and type I and II IFN responses (Figures 5C,D).

### Tumor-immune infiltration differences in the data showed the differences of immune landscape between the high-risk and the low-risk group

Using recently-developed immune infiltration algorithms to calculate the abundance of immune cells in different samples, we found that differences existed between the high-risk and the low-risk groups in immune scores and stromal scores; using the ESTIMATE algorithm, we also found estimate scores varied between the high-risk and the low-risk group. All data showed that the low-risk group exhibited a higher score (Figures 5F–J).

### The immune infiltration and tumor mutation analysis

**FIGURE 6:**
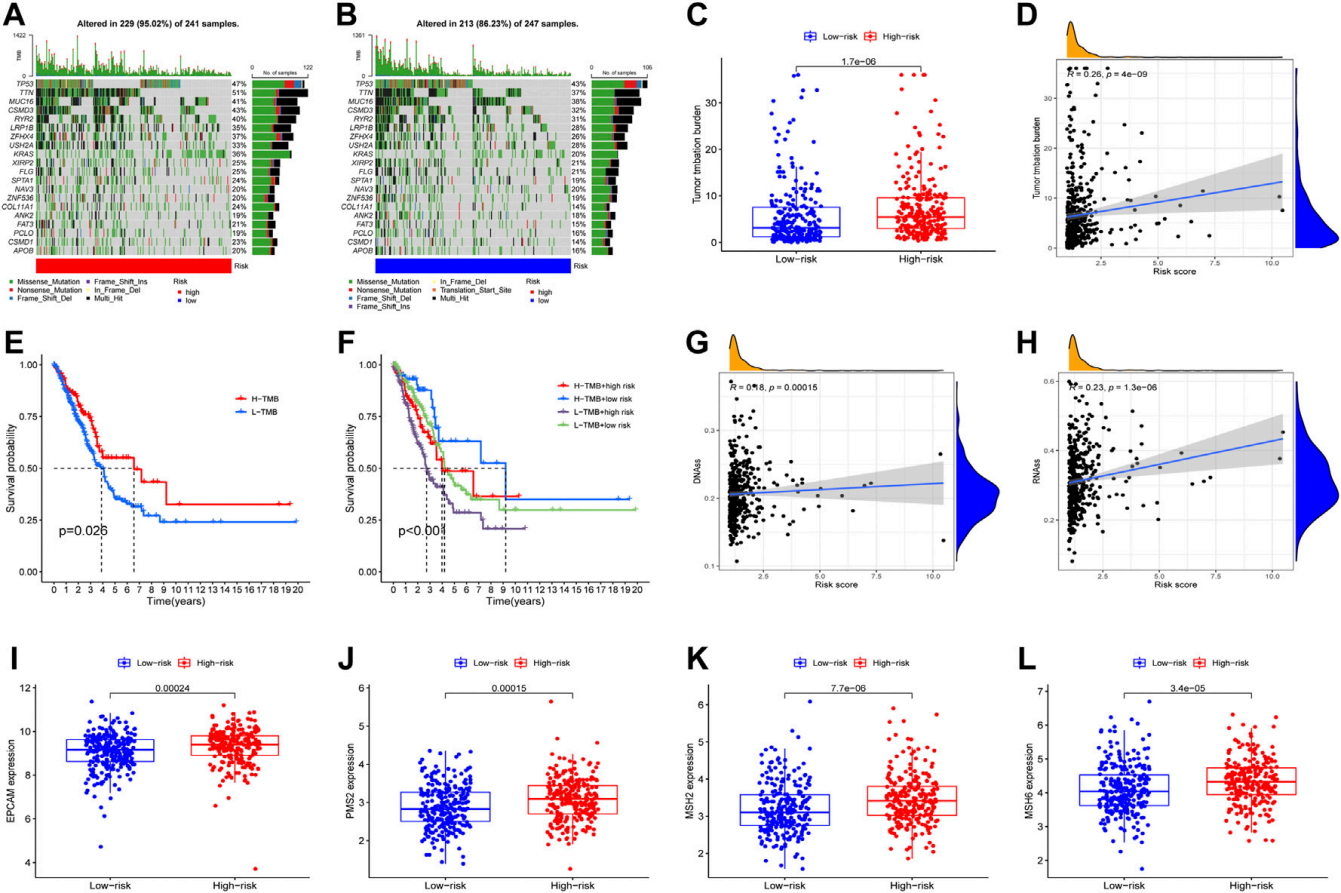
The relationship between risk score and mutation, tumor stemness, mismatch repair-related genes. Waterfall plots displayed the mutation information of top 20 genes with high mutation frequency in high-risk group **(A)** and low-risk group **(B)**. The difference of TMB between high-risk and low-risk groups **(C)**. The correlation of TMB and risk score **(D)**. Survival analysis of LUAD patients with different level of TMB and TMB combing with risk score **(E,F)**. The difference of tumor stemness index (RNAss and DNAss) between high-risk and low-risk groups **(G,H)**. The difference of mismatch repair-related genes expression between high-risk and low-risk groups **(I–L)**.

Tumor mutation analysis was subsequently conducted, and the most frequently mutated genes in the high-risk and the low-risk group were generally similar, with the most frequently mutated gene being TP53. The 10 genes most frequently mutated both in the high-risk group and the low-risk group were (listed in descending order): TP53, TTN, MUC16, CSMD3, RYR2, LRP1B, ZFHX4, USH2A, KRAS, XIRP2 (Figures 6A,B). Not surprisingly, the high-risk group carried significantly more mutation burden than the low-risk group (Figures 6C,D). Tumor mutation burden was subsequently analyzed combined with the risk scores and we found the group with a higher mutation rate received better clinical outcomes, and the clinical outcomes of patients in the L-TMB+high risk group were significantly poorer than other groups, compared with the H-TMB+low risk group which ranked best as regards the clinical outcomes (Figures 6E,F), and we found that the high-risk group was associated with higher mutation rate and higher tumor-stemness scores (Figures 6G,H). EPCAM PMS2, MSH2, and MSH6 expression were also compared between the low-risk group and the high-risk group, and all showed higher expression levels in the high-risk group (Figures 6I–L).

**FIGURE 7:**
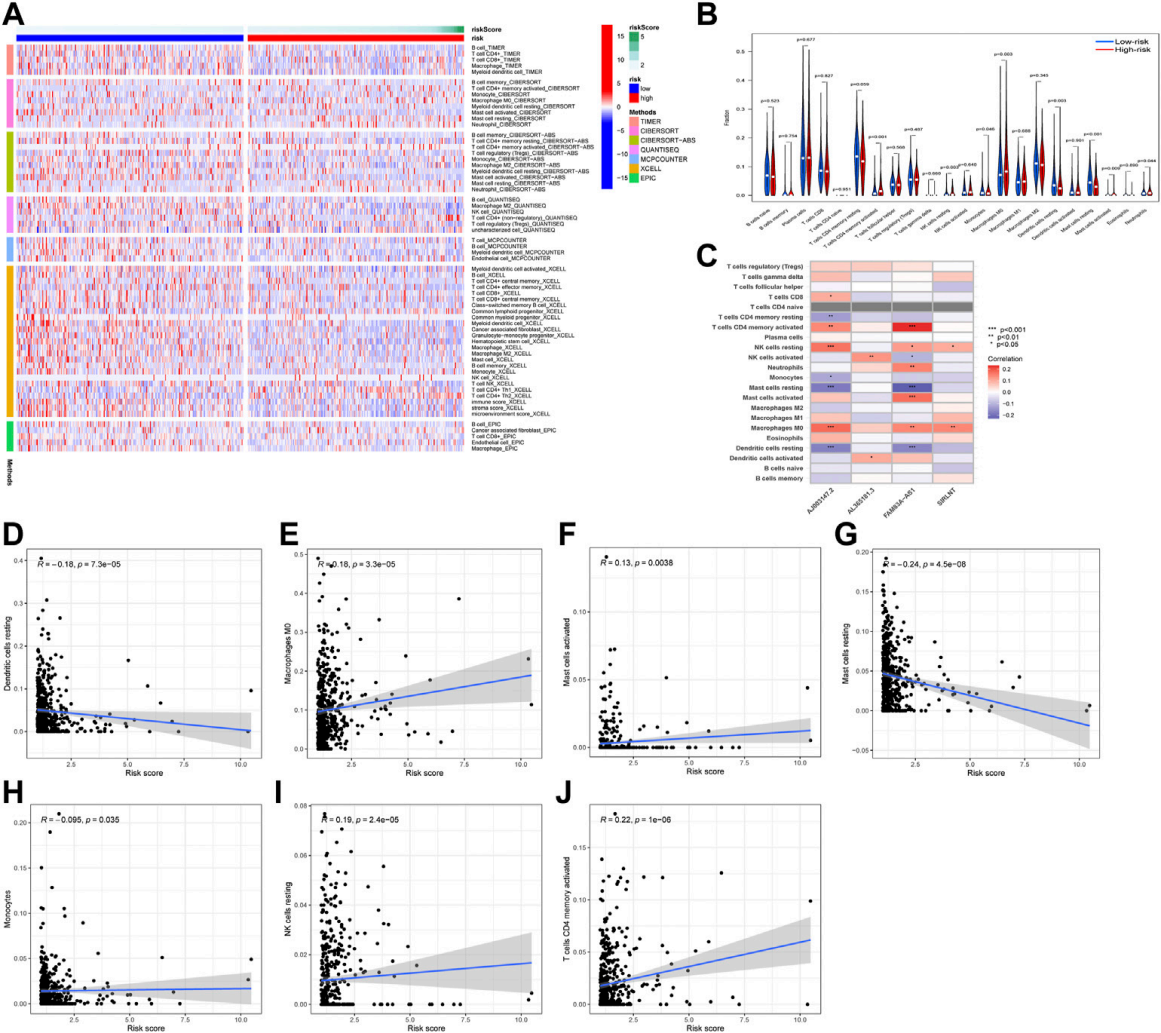
Analysis of immune cell infiltration. The immune landscapes of high-risk and low-risk groups **(A)**. The difference of immune cell between high-risk and low-risk groups **(B)**. The association of 4 NETs-associated lncRNAs expression and immune cell infiltration **(C)**. The association of risk score and immune cell infiltration **(D–J)** (* *p* < 0.05, ** *p* < 0.01, *** *p* < 0.001).

After applying multiple algorithms to calculate the immunological differences, we constructed an immune infiltration heatmap, and the results were shown to include the different results in immune infiltration (Figures 7A,B). Furthermore, the correlation between the infiltration and expression of every single gene was depicted using a heatmap (Figure 7C). We found that the risk score was correlated to the level of macrophages, dendritic cells, mast cells, NK cells, and T-cells (Figures 7D–J).

### Immune checkpoint and immunophenoscore analysis

**FIGURE 8:**
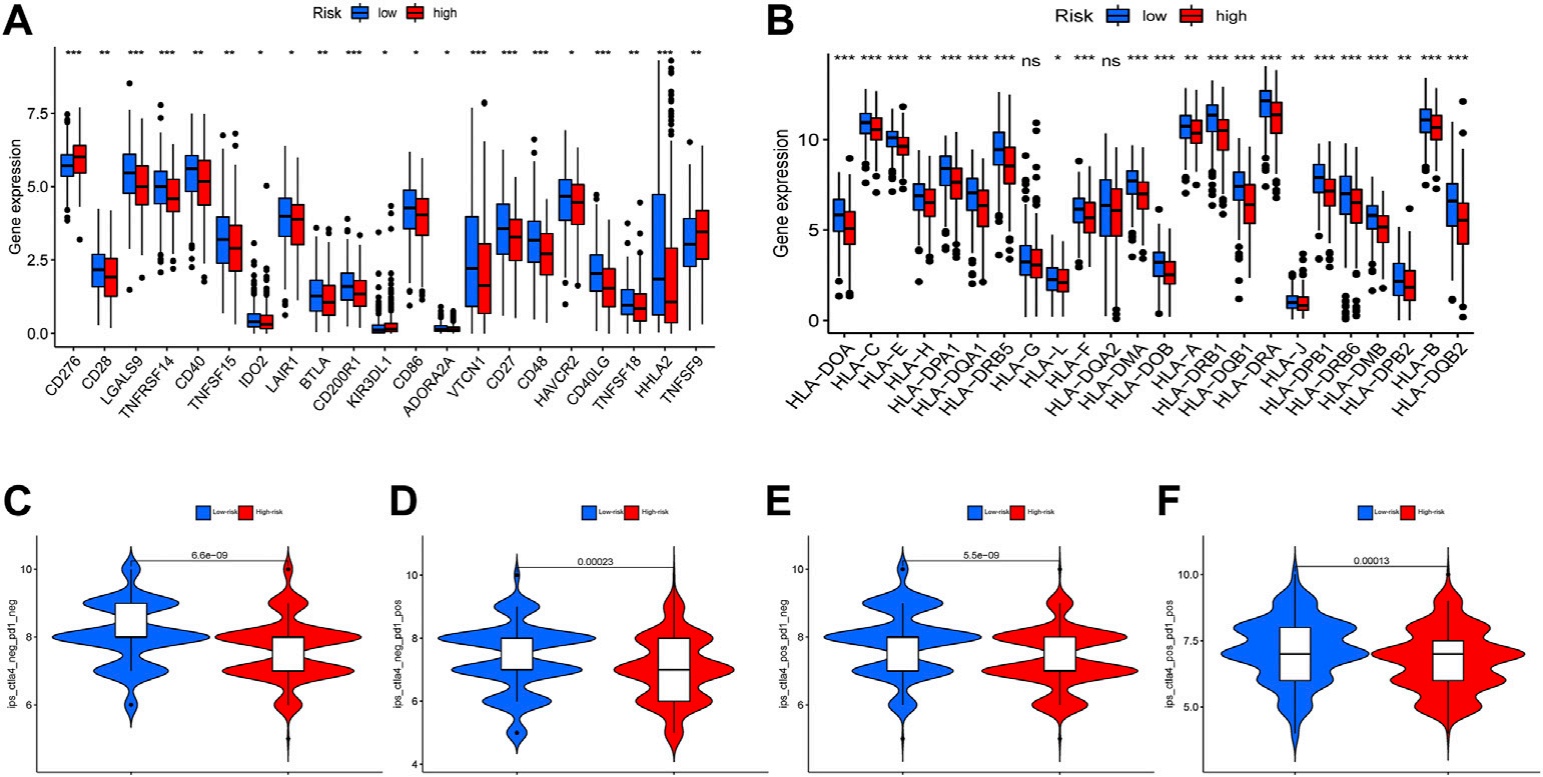
The correlations between risk score and immune checkpoint, immunephenoscores (IPS). The difference of immune checkpoint related genes **(A)** a and HLA-related genes **(B)** were showed in boxplot between high-risk and low-risk groups. The differences of IPS in patients with different risk are shown **(C–F)** (ns, not significant, * *p* < 0.05, ** *p* < 0.01, *** *p* < 0.001).

Immune checkpoints are a class of immunosuppressive molecules that are expressed on immune cells and regulate the degree of immune activation, and they play an important role in preventing the occurrence of autoimmunity (Zhai et al., 2021). Immune checkpoint genes were analyzed between the high-risk group and the low-risk group. The differences in the expression were quantified and depicted (Figure 8A). Although the immune checkpoint inhibitors like anti-CTLA4 monoclonal antibodies and anti PD-1/PD-L1 antibodies have been used clinically and improved patients’ outcomes, there are also other factors like human leukocyte antigens (HLAs) that affect the sensitivity to immunotherapy. Hence, we investigated the expression of HLA molecule family across the high-risk and the low-risk groups, and found that most of the HLA genes showed higher expression in low-risk groups (Figure 8B). Moreover, we re-analyzed the IPS z-scores across CTLA4^−^PD1^-^, CTLA4^−^PD1^+^, CTLA4^+^PD1^-^, and CTLA4^+^PD1^+^ groups, and found that in all subgroups, IPS scores were lower in the high-risk groups (Figures 8C–F).

### Drug sensitivity analysis

**FIGURE 9:**
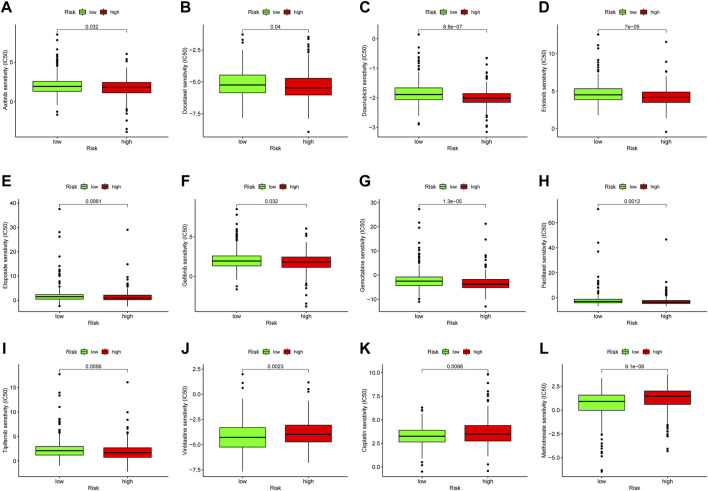
Drug sensitivity analysis. The IC50 of Axitinib, Erlotinib, Doxorubicin, Bortezomib, Gefitinib, Gemcitabine, Paclitaxel, Tipifarnib, Vinblastine, Vinblastine, Cisplatin and Methotrexate was analyzed in high-risk and low-risk groups **(A–L)**.

The study of the sensitivity of different groups of patients to chemotherapy or targeted-therapy drugs can provide help for the formulation of future treatment regimens. Using half maximal inhibitory concentration (IC_50_) as the index for the antitumor potency of the drugs, we investigated the differences in drug responsiveness between the high-risk and the low-risk groups. The high-risk group was more sensitive to antitumor drugs like Axitinib, Erlotinib, Doxorubicin, Bortezomib, Gefitinib, Gemcitabine, Paclitaxel, Tipifarnib, and Vinblastine (Figures 9A–I). Those antitumor drugs were potentially more capable of inhibiting high-risk uveal melanoma with relatively minor dosage. Conversely, drugs like Vinblastine, Cisplatin and Methotrexate exhibited lower antitumor efficiency in high-risk groups (Figures 9J–L).

## Discussion

As a lethal disease with a poor survival rate, the treatment of lung adenocarcinoma has become a heated topic in clinical oncology for decades. Despite the therapeutic advances based on progress in molecular biology and tumor immunology, the survival rate and efficiency of therapy are still not satisfactory (Relli et al., 2019; Anichini et al., 2020).

Neutrophil Extracellular Traps (NETs), a mechanism that is an indispensable part in innate immunity, are also involved in cancer progression and has been an emerging hotspot in recent years (Demkow, 2021). Accumulating pieces of evidence has shown that NETs could arouse dormant cancer cells, causing the unstrained growth and the metastasis of malignant tumors (Demers and Wagner, 2013). Also, NETs are believed to play a key role in the immune microenvironment of tumors. The close correlation between cancer cell and the co-localization between the tumor cells and NETs has also been discovered recently, which is believed to have a positive effect on the progression of the tumor (Masucci et al., 2020).

Although many molecular mechanisms have been found to take part in the pathogenes is of LUAD, little has been found about the mechanisms related to NETs. Recent pan-cancer analyses have figured out a prognostic signature related to NETs, which include the LUAD. However, to the best of our knowledge, a NETs-related signature has not been constructed for LUAD patients (Zhang et al., 2022).

Therefore, inspired by the recent discoveries of NETs’ oncogenic properties, we used TCGA to construct a NETs-related model based on the RNA-seq data. In our study, 469 NETs-related lncRNAs in total were identified as NETs-associated lncRNA lncRNAs. According to the univariate cox regression, 10 NETs-related lncRNAs were filtered and we separated the patients into 2 subgroups: cluster 1 and cluster 2. We found some differences between the 2 clusters that are related to survival and tumor immunity. The abundance of B cells memory and NK cells resting was significantly higher in cluster 2. NK cells are cytotoxic lymphocytes with direct killing effect in the innate immune system, participate in natural and adaptive immunity, and are the first line of defense for anti-tumor immunity (Valipour et al., 2019; Bald et al., 2020). ESTIMATE score, PD-L1expression, CTLA4 expression and IPS score are lower in cluster 2, which means that patients in cluster 2 have lower immunogenicity. So it can be applied to forecast the immunotherapeutic effect of LUAD patients.

Next, we used the LASSO regression method to reduce the variable to four, reducing over-fitting while strengthening the clinical significance of the model. The results showed the signature we constructed was of strong clinical relevance, and could effectively predict patients’ outcomes. Calibration is used to describe the accuracy of a model to predict the probability of individual clinical outcomes. In practical application, it is usually characterized by calibration curve. The calibration curve shows the deviation between the predicted value of the model and the actual value, which is another way to test the prediction ability of the model (Gittleman et al., 2020). Similar to the results of other studies (Liu et al., 2021b; Cui et al., 2022), the calibration curve showed that the observed OS ratios in 1, 3 and 5 years were in good agreement with the predicted ratios.

Also, we discovered the differences between the immune cell infiltration, and immune checkpoint analysis, and found out that the results are related to the infiltration of various types of immune cells, such as macrophages, T cells, and NK cells. In addition, immune infiltration affects the survival rate of tumor patients (Riquelme et al., 2019). The results indicate that different types of immune cells are correlated to the signature, and the sensitivity to immunotherapy varied between the low-risk group and the high-risk group, further demonstrating its clinical significance. Low TMB is associated with low immune infiltration, which means a poor immune response (Hu et al., 2021). HLA alleles have been shown to stratify tumor patients with high accuracy (Callahan et al., 2018). This may be the mechanism of the difference in immunogenicity between the two groups. Moreover, it is also recommended to perform RNA-seq in clinically harvested samples, calculating immunephenoscores (IPS) and immune infiltration scores and to validate the relevance with our previous model (Chen et al., 2022a). Then, we analyzed the drug sensitivity of the two risk groups, providing targeted guidance for LUAD patients to choose treatment drugs.

Of all 4 NETs-related lncRNAs that are involved in the prognostic signature, it has been proved that lncRNA-FAM83A-AS1 could promote tumor progression in lung adenocarcinoma, *via* promoting the HIF-1α/glycolysis axis (Chen et al., 2022b). Our study further validated its robust capability as a biomarker in lung adenocarcinoma. lncRNA SIRLNT was recently discovered as a tumor promoter in breast cancer, by regulating the miR-4766-5p (Liang et al., 2018). However, whether those lncRNAs could act as a tumor promoter in LUAD requires further investigation.

However, due to the limitations of bioinformatic methods, we were only capable to find the correlations between the scores and immune phenotypes. Further studies are needed to reveal the mechanisms that lie within, including *in vitro* and *in vivo* studies regarding the molecular mechanisms. Also, the interactions between the lncRNAs in the signatures we constructed still require further investigation.

## Conclusion

Above all, the risk model we constructed was strongly correlated to the NETs properties and could predict patient outcomes. Besides, whether the risk model was correlated to the sensitivity of immunotherapy still requires further investigation. Therefore, *in vitro* and *in vivo* experiments regarding lung adenocarcinoma are urgently needed to test the possible target lncRNAs and their oncogenic mechanisms.

## Data Availability

The datasets presented in this study can be found in online repositories. The names of the repository/repositories and accession number(s) can be found in the article/Supplementary Material.
