# Supplementary material for: A neutrophil extracellular traps-associated lncRNA signature predicts the clinical outcomes in patients with lung adenocarcinoma
Source: Front Genet. 2022 Nov 7;13:1047231. doi: 10.3389/fgene.2022.1047231 (PMC9676361; doi:10.3389/fgene.2022.1047231)
Supplement: Supplementary file 1 [file Table1.docx]

| Covariates | Type | Total | Test | Train | Pvalue |
| --- | --- | --- | --- | --- | --- |
| Age | <=65 | 229(47.91%) | 109(45.42%) | 120(50.42%) | 0.3157 |
| Age | >65 | 249(52.09%) | 131(54.58%) | 118(49.58%) |  |
| Gender | 0 | 256(53.56%) | 124(51.67%) | 132(55.46%) | 0.4592 |
| Gender | 1 | 222(46.44%) | 116(48.33%) | 106(44.54%) |  |
| Stage | 1 | 258(53.97%) | 129(53.75%) | 129(54.2%) | 0.9016 |
| Stage | 2 | 118(24.69%) | 57(23.75%) | 61(25.63%) |  |
| Stage | 3 | 80(16.74%) | 43(17.92%) | 37(15.55%) |  |
| Stage | 4 | 22(4.6%) | 11(4.58%) | 11(4.62%) |  |
| T | 1 | 163(34.1%) | 79(32.92%) | 84(35.29%) | 0.7556 |
| T | 2 | 253(52.93%) | 126(52.5%) | 127(53.36%) |  |
| T | 3 | 44(9.21%) | 25(10.42%) | 19(7.98%) |  |
| T | 4 | 18(3.77%) | 10(4.17%) | 8(3.36%) |  |
| N | 0 | 315(65.9%) | 155(64.58%) | 160(67.23%) | 0.462 |
| N | 1 | 91(19.04%) | 48(20%) | 43(18.07%) |  |
| N | 2 | 70(14.64%) | 37(15.42%) | 33(13.87%) |  |

Table S1. The statistical data of clinical characteristics of the three sets.
